# Ultraviolet and Infrared luminescent Au-rich nanostructure growth in SiO_2_ by burrowing and inverse Oswald ripening process

**DOI:** 10.1038/s41598-019-51169-x

**Published:** 2019-10-18

**Authors:** D. P. Datta, A. Chettah, Arpan Maiti, B. Satpati, P. K. Sahoo

**Affiliations:** 1 School of Physical Sciences, National Institute of Science Education and Research, Bhubaneswar, HBNI, Jatni, 752050 India; 2 Basic Sciences and Humanities Department, Silicon Institute of Technology, Bhubaneswar, 750024 India; 3 LGMM Laboratory, Functional Materials Group, Université 20 Août 1955-Skikda, BP 26, 21000 Skikda, Algeria; 4 Surface Physics and Material Science Division, Saha Institute of Nuclear Physics, Kolkata, 700064 India

**Keywords:** Materials science, Nanoscale materials

## Abstract

We study the evolution of nanoparticle morphology through successive stages when Au-Si bilayer on SiO_2_ is irradiated with 500 keV Xe-ions and resulting luminescence in the UV, Visible and infrared range. An array of nanoscale island morphology is developed on the silica surface in the initial stage of evolution which undergoes gradual burrowing in the silica matrix accompanied by elongation of large ones in the direction of incident ions under cumulative ion irradiation. Burrowing is found to occur in order to minimize the surface free energy of the nanoparticles. Numerical simulation based on the unified thermal spike model shows formation of molten latent tracks due to ions energy release which drive the dewetting of the metal layer and further give mobility to nanoparticle atoms leading to burrowing in the later stage of evolution and elongation of large nanoparticles. Satellite nanoparticles are observed to grow around Au nanoparticles embedded in silica through nucleation of Au atoms dissolved in the matrix by incident ions. The varying diameters of the Au satellite nanoparticles seem to result in luminescence in the UV and infrared range. The observed structure may find application in surface enhanced Raman scattering, catalysis, and LEDs.

## Introduction

Unique properties of nanoscale structures of metals, semiconductors, or their composites, originating from large surface to volume ratio, charge confinement in low dimension, etc., make them key components in present and future applications of nanotechnology^1–8^. Promising progress in plasmonics, photonics, optoelectronics, catalysis, sensors, or bioscience have been achieved through characteristic properties of nanoparticles, for instance, surface Plasmon resonance (SPR), i.e. strong absorbance of radiation at particular wavelength due to collective oscillation of conduction electrons in metal nanoparticles or enhanced electric field in the vicinity of a metal nanoparticles^5,7^. In the contexts of research on application of nanoparticles, Au nanoparticles receive particular attention for their potential applications. Catalytic activity of Au nanoparticles is utilized for growth of nanowires and nanotubes^9–11^. SPR and local field enhancement of Au nanoparticles are highly promising for surface enhanced Raman scattering (SERS)^12,13^ plasmonic waveguides, photocatalysis, or biosensing^14–17^. Studies of nanoscale behaviour also indicate that the functionalities of nanoparticles can be significantly enhanced in case of metal-semiconductor nanocomposites^5–8^. A nanoscale composite of Au with any semiconductor material, therefore, holds highly promising possibility towards further improved physical and chemical properties. This fact motivated significant research effort towards synthesis of varied types of composite nanostructures with an emphasis on controllability of their shape and size, as well as exploration of their novel properties. Among the physiochemical techniques for synthesis of nanoscale structures, dewetting of a metal film by irradiation of energetic ion beam has offered single step self-organized processes^18–24^. Dewetting of a metal films on a non-wetting surface is a self-organized process where the continuous film ruptures due to ion irradiation and gradually transforms into an array of nanodots on the substrates^18–23^. Ion irradiation induced dewetting is particularly attractive process because it offers control over a number of experimental parameters like ion energy, fluence, angle of incidence, etc. and the dimension and distribution of the nanostructures are function of these experimental parameters^18–22^. Moreover, in case of multi-layered structure, ion irradiation results in mixing of materials across the interface and formation of phases. The sputtering due to ion energy deposition during ion induced dewetting also contributes to nanostructure evolution. Thus, ion irradiation technique couples the nanostructure evolution due to dewetting with ion induced mixing and sputtering, opening up a promising route towards single step self-organized synthesis of composite nanodots on materials surface^25–28^. In our studies, we have shown that ion-irradiation effectively transforms a Au-Si or Au-Ge bilayer to a nanoparticle array on SiO_2_ surface^25–28^. The initial stage of the evolution triggered by the ion irradiation was related to ion-induced dewetting of the metal layer accompanied by sputtering and mixing. However, the detailed structural evolution of the nanoscale morphology up to high ion fluence regime, and exploration of the resulting properties such as luminescence, is yet to be understood. In this study, we demonstrate ion-induced burrowing of the nanoscale array morphology due to melting and material flow in the ion track with cumulative ion irradiation, elongation of large nanoparticles with burrowing, formation and growth of satellite nanoparticles out of dissolved atoms and UV-visible-infrared range luminescence of the nanoparticle morphology.

## Experimental

The depositions of Si and Au thin films on SiO_2_ were carried out using e-beam evaporation in a high vacuum deposition chamber having a base vacuum of 2 × 10^−7^ mbar. Commercially available thermally grown SiO_2_ (thickness 300 nm) on Si were used as substrate. The thicknesses of the deposited films were measured by a quartz crystal monitor. 6 nm Si thin films were deposited on the SiO_2_ surface followed by deposition of 6 nm Au films. The deposited samples were irradiated by 500 keV Xe^2+^ ions at the Low Energy Ion Beam Facility at IUAC, New Delhi. For a comparison with the evolution of the bi-layer structure, ion irradiations were also carried out on 6 nm thin Si films deposited on SiO_2_. During irradiation, the incident ion-flux was kept constant around 1 μA cm^−2^ and the beam was scanned over the irradiated area in a raster pattern to achieve homogeneous irradiation. Au-Si nanostructure morphology developed by ion irradiation was investigated using scanning electron microscopy (SEM).The photoluminescence (PL) spectra from the nanostructures were recorded at room temperature using the 325 nm line of a He-Cd laser as primary excitation source. The cathodoluminescence (CL) spectra were also obtained at room temperature using a 30 keV electron beam in an SEM. The cross-sectional transmission electron microscopy (XTEM) of selected samples were carried out in a field emission gun based 300 keV FEI TecnaiG^2 ^S-Twin.

## Results and Discussion

**Figure 1:**
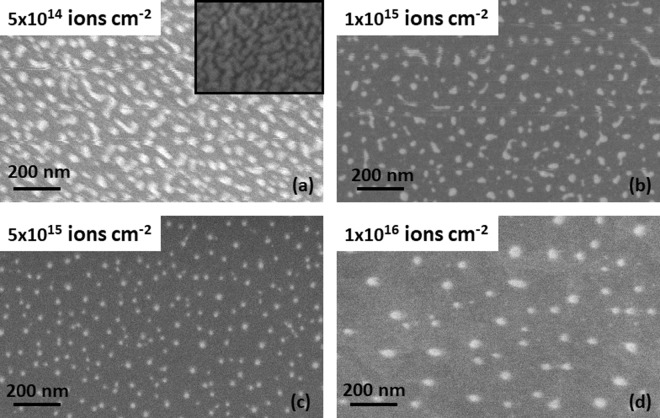
SEM images of nanoparticle morphology developing on SiO2 with increasing ion irradiation on the Au-Si bi-layer. Ion fluences are indicated on the images.

Nanodot-array morphology gradually evolves on SiO_2_ surface as ion energy is deposited in the bilayer upon irradiation. The inset of Fig. 1(a) shows the morphology of as-deposited film, which comprises of randomly shaped grains separated by fissures. Extended structures coexist with circularly shaped island morphology in the initial stage of morphology evolution as shown in Fig. 1(a,b), corresponding to flounces of 5 × 10^14^ and 1 × 10^15^ ions cm^−2^, respectively. Both areal densities of islands and number of extended structures decrease with increased ion fluence [see Fig. 1(b)]. Array of circular dots forms at higher fluences as shown, for instance, at fluences 5 × 10^15^ and 1 × 10^16^ ions cm^−2^, in Fig. 1(c,d). It should be mentioned here that we performed the compositional analysis of the nanostructures using XPS, which revealed that Si is present in the Au-rich nanodots formed under irradiation and AuSi phase is formed^27^.

**Figure 2:**
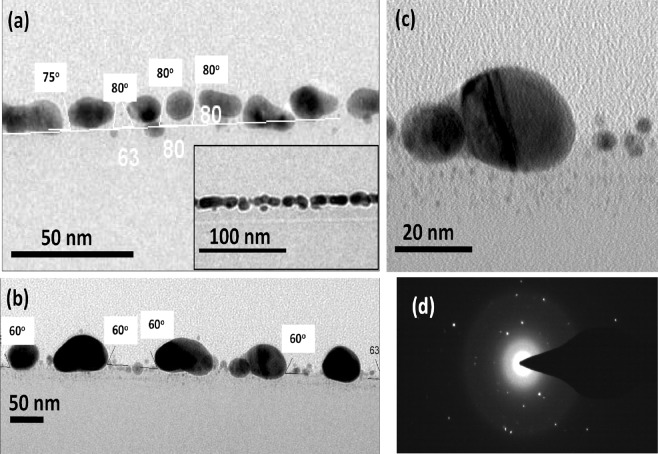
XTEM images showing the nanoparticle morphology corresponding to as deposited sample (**a**), ion fluence of 5 × 10^14^ ions cm^−2^ (**b**), a magnified image of a single nanoparticle (**c**), and SAED image from region containing the nanoparticles (**d**). The inset in (**a**) shows the morphology of the as-deposited sample. The contact angle of the Au-rich nanoparticles with the SiO_2_ surface is indicated in **(a**,**b**).

**Figure 3:**
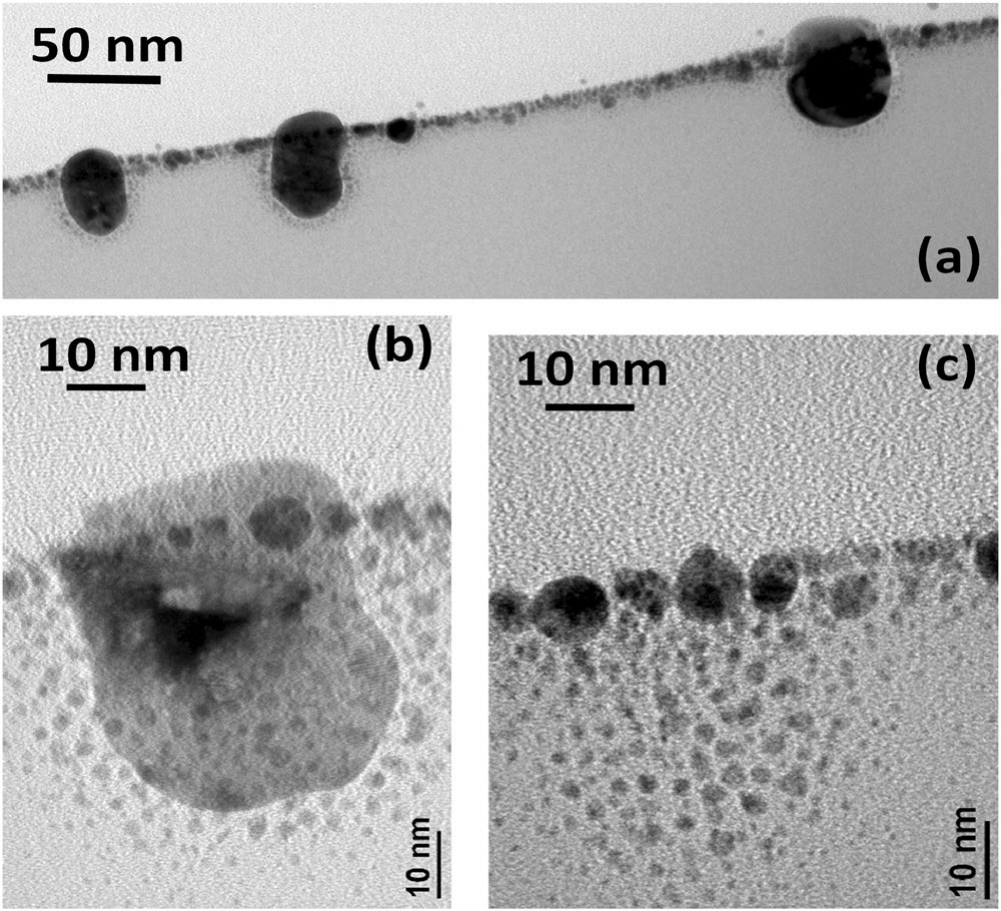
(**a**) XTEM images showing the elongated nanoparticles undergoing burrowing and growth of satellites at ion fluence of 1 × 10^16^ ions cm^−2^ (**b**) a magnified image of a single elongated nanoparticle with satellites (**c**) A magnified view of the region in between the elongated nanoparticles showing growth of secondary nanoparticles below the primary nanoparticles on surface.

Figures 2 and 3 show the XTEM images of the as deposited sample and samples irradiated to the fluences of 5 × 10^14^ ions cm^−2^ and 1 × 10^16^ ions cm^−2^. The details of the structural evolution, from thin film to nanodots at initial stage of irradiation, and development of embedded nanostructures at higher ion fluences become clear from the XTEM images. The XTEM image of the as-deposited sample is shown in Fig. 2(a). The structure observed in the image is consistent with the morphology observed in the top-view SEM image in the inset of Fig. 1(a). Figure 2(b) is a representative image of the sample irradiated to the fluence of 5 × 10^14^ ions cm^−2^. The corresponding SEM image is in Fig. 1(a). The peak of the particle diameter distribution was determined from SEM contrast to be around 35 nm with a width around 15 nm^27^. The lateral dimensions of the nanostructures observed in the SEM image is consistent with the XTEM image of Fig. 2(b). Smaller nanoparticles of dimension 6–10 nm are also observed at the surface, in between the larger ones, which are not clearly resolved in the SEM images. XTEM image further reveals that the nanodots get partially buried in SiO_2_ matrix under irradiation. Close inspection also shows nucleation of smaller nanoparticles of dimension less that 1 nm in the SiO_2_ matrix underneath the large nanoparticles. This aspect is clarified in the higher magnification XTEM image of a single large nanoparticle in Fig. 2(c). Nucleation of nanoparticle is observed up to a depth of ~10 nm below the large nanoparticle. The selected area electron diffraction image (SAED) of the area containing the nanoparticles is shown in Fig. 2(d), where the spot pattern exhibits existence of crystallinity in Au nanoparticles, although deteriorated by ion induced defect formations. The XTEM images in Fig. 3 illustrate further embedment of the nanostructures into SiO_2_ at the highest fluence of 1 × 10^16^ ions cm^−2^ and modification in particle shape. In this regime, we observed a few elongated nanoparticles, having width 30–40 nm and length 40–50 nm. A comparison of the XTEM images of Figs 2(b) and 3(a) clarifies that the large nanoparticles (20–50 nm) get elongated while they also get burrowed in the SiO_2_ matrix. The inter-distance of the nanoparticles in the XTEM image (around 50–200 nm) is also consistent with the top-view SEM image of Fig. 1(d). Only the top portions of the elongated nanoparticles are protruding from the SiO_2_ matrix. Embedded portion of the nanoparticles are surrounded by smaller satellite nanodots of varying diameter within a distance of ~15 nm from the large nanoparticle surface. Further, the layer of smaller nanoparticles of 6–10 nm diameters is also observed in between these elongated ones. In Fig. 3(b), we show higher magnification image of a large nanoparticle. The diameters of the satellite nanoparticles surrounding the elongated ones is found to decrease as a function of distance from the primary nanoparticle. Close to the primary one, the satellite diameters are 2–3 nm whereas at the farthest region, diameters of the nanoparticles are below 1 nm. In Fig. 3(c), we show the representative XTEM image of a region between the elongated large nanoparticles. The top portions of the nanoparticles are protruding from the surface, similar to the elongated ones. In addition, a layer of nanoparticles have formed in the SiO_2_ matrix below the top layer. The diameter at the bottom part of the distribution is found to be less than 1 nm whereas the diameters are 2–3 nm near the surface. It appears that the sub-nm nanoparticles observed in Fig. 2(b) are growing with ion irradiation, whereas new nanoparticles nucleate at greater depth under irradiation. The important aspect that the top portions of the nanoparticles are protruding while the bottom part is embedded in SiO_2_ matrix make such nanoparticle array suitable for applications like SERS substrates^12,13^.

In order to get a theoretical understanding of observed structural evolution in terms of ion-energy deposition in the present system, we have performed a simulation of ion energy deposition by 500 keV Xe-ions in our deposited bilayer structure on SiO_2_. The simulation is carried out using the unified thermal spike model^29–31^. In fact, this model can be applied to account for the synergy between the elastic and inelastic thermal spikes. Recently it has been extended to multilayer systems^28^. More details are found in the previous paper^28^ for Au/Ge system irradiated with the same energy as in the present system (500 keV Xe ions). The main hypotheses in this model are:

(i) Both electronic and nuclear energy losses contribute to the target heating process along the ion path as described in the following set of coupled heat diffusion Eqs (1) and (2) for electronic and atomic subsystems, respectively^30,31^:
  1 $${C}_{e}\frac{\partial {T}_{e}}{\partial t}=\frac{1}{r}\frac{\partial }{\partial r}(r{K}_{e}({T}_{e})\frac{\partial {T}_{e}}{\partial r})+(\frac{\partial }{\partial x}{K}_{e}({T}_{e})\frac{\partial {T}_{e}}{\partial x})-g({T}_{e}-{T}_{a})+A(r,t),$$
  2 $${C}_{a}\frac{\partial {T}_{a}}{\partial t}=\frac{1}{r}\frac{\partial }{\partial r}(r{K}_{a}({T}_{a})\frac{\partial {T}_{a}}{\partial r})+(\frac{\partial }{\partial x}{K}_{a}({T}_{a})\frac{\partial {T}_{a}}{\partial x})+g({T}_{e}-{T}_{a})+B(r,t),$$
  where *C*_*e,a*_, *T*_*e,a*_, and *K*_*e,a*_ are the specific heat, temperature, and thermal conductivity of the electronic and atomic subsystems. *g(T*_*e*_ *−* *T*_*a*_) represents the electron phonon coupling term where *g* is the electron phonon coupling coefficient. A *g* value of 3 × 10^10^ W/cm^3^/K has been deduced for Au, from femto-second laser experiments^32^. A *g* value of 1.8 × 10^12^ and 1.25 × 10^13^ W/cm^3^/K has been used for Si^33^ and SiO_2_^34^ *A*(*r,t*) and *B*(*r,t*) are electronic and nuclear energy densities respectively^30,31^.
(ii) The thermal properties of the target materials, namely melting temperature, latent heat of fusion and evaporation, temperature dependence of lattice specific heat and thermal conductivity are taken from empirical values at equilibrium for bulk materials.
(iii) The time and space energy distribution on electronic and atomic subsystems, *A(r,t)* and *B(r,t)*, are very important in the calculations and were simulated using analytical functions from literature^29–35^. It is worth noting here that, for 500 keV Xe ions, *B(r, t)* contribution to the heating process is much higher than *A(r, t)* due the higher values of nuclear energy losses (*Sn*) as compared to those of the electronic energy losses (*Se*). Therefore, the choice of time (*τ*) and radius (*r*) of nuclear energy distribution^29^ is very decisive in our present case. Toulemonde *et al*.^29^ has tested the effect of the choice of both parameters and found that variation of the deposition time by a factor of 4 (between 5 × 10^−14^ and 2 × 10^−13^ s) causes only a 10% difference in the molten radius, while a 50% increase of *r*_0_ leads to a 50% variation in the molten radius.
(iv) Solid-liquid transformation or melting occurs when the energy per atom exceeds the energy per atom necessary for melting, *E*_*am*_, given by the following expression
  3 $${E}_{am}={\int }_{{T}_{initial}}^{{T}_{melting}}{C}_{s}(T)dT+{L}_{m},$$
  Here the first term represents the energy necessary to reach the melting temperature, where *C*_*s*_*(T)* is the lattice specific heat. *L*_*m*_ is latent heat of fusion. The melting energies per atom, *E*_*am*_, are: 0.43, 0.84, and 0.38 eV/atom for Au, Si and SiO_2_, respectively.
(v) For multilayer systems, the interface between materials is supposed to be consisting of a perfectly plane surface and no intermixed region exists as shown in Fig. 2.
(xvi) The electronic and nuclear energy loss are assumed to be independent of depth along each material, which is true when dealing with very thin layers as discussed previously^28^ (in the present case Au and Si are only 6 nm each).

**Figure 4:**
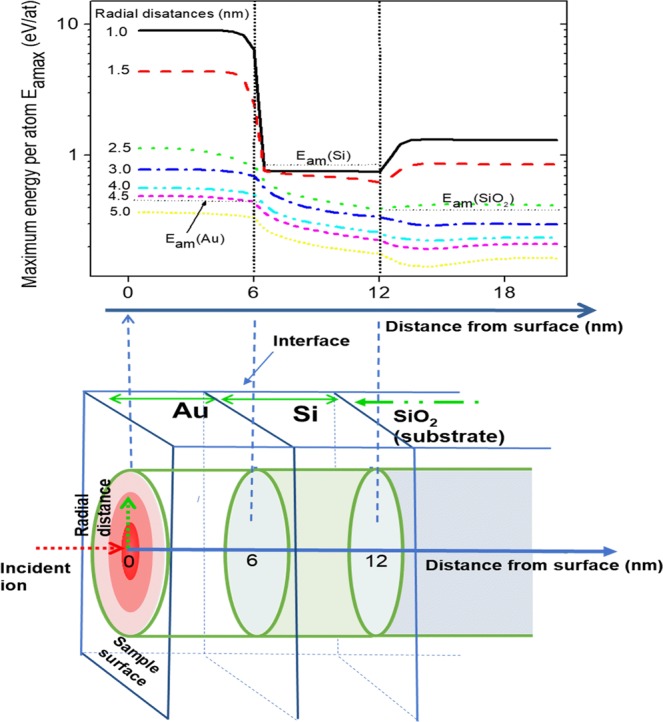
Evolution of the maximum energy per atom, E_amax_,for different radial distances from the ion path in Au(6 nm)/Si(6 nm)/SiO2(substrate) target under 500 keV Xe ion irradiation. The horizontal dashed dot lines indicate the melting energy per atom E_am_(Au), E_am_(Si) and E_am_(SiO_2_) for Au, Si and SiO_2_, respectively. The vertical dashed dot lines show theAu/Si and Si/SiO_2_ interfaces. The schematic at the bottom of the figure shows the multilayer structure as well as the cylindrical symmetry of the energy deposition of an incident ion on both electronic and atomic subsystems. The energy per atom (or lattice temperature) is the highest at the impact position and gradually decreases with radial distance, as illustrated with the red colour nuance.

The calculation code solves numerically the electronic and atomic heat diffusion Eqs (1) and (2), and provides the space and time evolution of energy per target atom, which is equivalent to temperature, as the main output. The calculations were performed in superheating scenario^36^. In Fig. 4 we show the evolution of the energy per atom versus time for different radial distances from the ion track for 500 keV Xe ion irradiation in Au(6 nm)/Si(6 nm) bilayer on SiO_2_ substrate. The horizontal black lines show melting energy per atom *E*_*am*_ for each of the materials. The results clearly show that in our experimental situation a molten zone is formed in Au layer and SiO_2_ around the ion track. However, the energy per atom is slightly lower that that required for melting for the Si layer. The radii of the molten zones are 4.5 nm and 2.5 nm for the Au and SiO_2_ layers, respectively. However, if we consider the phenomenon of melting point depression with the reduction of the size of the material to few nanometres, the energy required for melting might be much lower than that for bulk materials. Consequently, even Si with 6 nm thickness might thought of, qualitatively, to exhibit melting after 500 keV Xe ions irradiation. Further, the bilayer is converted into Au-rich nanoparticles in the initial stage. Subsequently, melting can be assumed to take place along the ion track in both the nanoparticle and SiO_2_ matrix, although the redii of molten zones can be different. Thus, it can be inferred that the dewetting of the Au layer leading to nanoscale islands and consequent structural transitions is largely influenced by formation of molten zones due to ions energy deposition. Below we discuss the observed evolution in terms of the results of the simulation.

The ion-induced dewetting of process of the deposited bi-layer in the initial stage was discussed previously^27^. However, the important issue of melting of the materials along the ion track in the Au and Si layer had not been addressed through quantitative theoretical estimation, which we have shown here. The formation mechanism of nanoscale extended structures/islands from a Au-Si bilayer in the initial stage is primarily the ion-beam-induced dewetting of the top metal layer, which is driven by ion energy deposition^18–22^. The evolution process is further influenced by ion beam induced mixing of Au and Si, and sputter-erosion of materials from the surface^27,28^. The simulation above has clearly shown that energy loss of incident ions within the bilayer leads to formation of a localized molten zone along the ion track, which persists for a time of the order of picoseconds. On the other hand, the surface tension of molten Au also differs from that of solid state. The surface tension gradient and pressure gradient within the molten zone induce flow of the material from the molten track to the surface of the top layer^18,19^. Overall result of material flow is similar to the dewetting of a liquid layer on a non-wetting solid surface, which results in rupture of a continuous liquid film. Array of extended structures and islands are developed from a metal layer by nucleation of dry patches in the layer^18–20^ or by amplification of thermally generated amplitude fluctuations^21,22^. The extended structures subsequently evolve into regular circularly shaped particles/dots by mechanism such as Rayleigh instability^37^. At the same time, continuing ion irradiation leads to burrowing of the nanoparticles.

Burrowing of nanoparticles into a substrate under ion irradiation has been observed in previous studies, for instance, by Hu *et al*. for 800 keV Kr-ion irradiation on Pt thin film on SiO_2_ and Al_2_O_3_^38^, and by Prakash *et al*. for 150 keV Ar-ion irradiation on Au thin film deposited on polymer matrix^23,39^. Burrowing of nanostructures on a substrate surface can be understood by the thermodynamic instability of the structures. When the interface energy of a metal nanoparticle and the substrate is smaller than the surface free energy of the metal in ambient, the nanoparticle is thermodynamically unstable. If the metal atoms get enough mobility then the nanostructures can get buried in the substrate in order to minimize the free energy^23,38–41^. Thermal treatment or ion irradiation greatly enhances atomic mobility within the nanostructures. As a matter of fact, we have shown above through numerical simulation of the present experimental situation that ion irradiation induces formation of ion track of a few nm radius which remains effectively in molten state for picosecond time. It has been observed that estimated ion track radius is ~4.5 nm in Au and 2.5 nm in SiO_2_ [see Fig. 4]. To associate the observed burrowing with this theoretical origin, we determine the contact angles of the nanoparticles on SiO_2_ from the XTEM images. If a particle makes a contact angle θ_c_ with a substrate, then the local equilibrium condition for the particle is *γ*_*p*_ + *γ*_*ps*_ *cos θ*_*c*_ = *γ*_*s*_, where *γ*_*p*_ and *γ*_*s*_ are the surface free energies of the particle and the substrate, respectively, and *γ*_*ps*_ is the free energy of the interface. The angle of the as-deposited thin film comprising of nano-islands are found to be 75°–80°, on the average, as shown in Fig. 3(a), so the contact angle is π - 75° = 105°. The free energy of Au at melting temperature^40^ is 1.39 J m^−2^ and *γ*_*s*_ for SiO_2_ is 0.31 J m^−2^ at melting point^38^. Using these values and the contact angle *θ*_*c*_, the interface energy *γ*_*ps*_ is found to be 0.67 J m^−2^, which is much less that *γ*_*p*_ for Au. For ion irradiated Au nanoparticle in SiO_2_, the interface energy was found to be 0.1–0.2 J m^−2^ by Rizza *et al*.^42^, which is comparable to our estimated value. It should be mentioned here that the surface free energy of free Au nanoparticle has been found to be 8.78 J m^−2 ^^39^, which is much greater than that of the bulk Au. If this value is considered, the surface energy of Au nanoparticle embedded in SiO_2_ will be significantly lower^24,39^. It is further observed from Fig. 3(b) that the angle between the partially embedded Au and the substrate surface decreases to 60°, making the contact angle 120°, which indicates irradiation induced decrease in interface energy. So the observed burrowing of the Au-Si nanoparticles can be attributed to reduction in surface free energy of the nanostructures in SiO_2_, because metal atoms in the nanoparticles are mobile in the molten ion track.

Elongation of spherical metal nanoparticles embedded in insulators, in the direction of ion irradiation, is well studied in case of Swift heavy ion irradiation where electronic energy loss dominates the ion-target interaction^43–45^. It is interpreted as a result of flow of metal atoms of the nanoparticles for in-plane stress release. Material flow is induced in a molten zone formed around the ion track through the metal nanoparticles and transition of spherical nanoparticles to elongation shape takes place by consecutive effect of many ions. Elongation of spherical nanoparticles in low ion energy regime where nuclear energy loss gives a significant contribution to energy deposition was however observed, for instance, in case of Au nanoparticles in SiO_2_ under 3 MeV Au ion irradiation^46^. In the present study, we see that elongation sets in subsequent to onset of burrowing of the nanoparticles [Figs 2(b) and 3(a)]. Our numerical simulation demonstrates molten zone formation around the ion track in the Au as well as in SiO_2_, with diameters 4.5 nm and 2.5 nm, respectively. Therefore material flow can be induced in the molten zone leading to elongation of the nanoparticles and this process may occur parallel to burrowing.

Ridgway *et al*. reported^45^ that, under swift heavy ion irradiation on metal nanoparticles embedded in SiO_2_, spherical nanoparticles having diameter above a critical value get elongated, whereas nanoparticles having diameter below may get dissolved. The critical value is related to ion track diameter in SiO_2_ and for Au nanoparticles it is within 10–11 nm. In our experiment, we observe the lateral dimensions of the elongated Au nanoparticles are much larger, 30–40 nm. Further, diameter of molten ion track in SiO_2_ (2.5 nm) is smaller than that in Au(4.5 nm).However, the fact that smaller nanoparticles having diameters 6–10 nm [Fig. 3(a,c)] does not undergo elongation, was also observed by Ridgeway *et al*.^45^. It is also to be noted that the ion fluence applied in the present experiment, where nuclear energy loss dominates, is two-order-of-magnitude higher than that applied in swift heavy ion experiments. We assume that in low energy ion irradiation, elongation of nanoparticles proceeds in a cumulative by increasing no of ions going through the nanoparticles but is still related to material flow in the molten ion track in Au and SiO_2_. Since the larger nanoparticles (30–40 nm diameter) have a larger in-plane areal density compared to smaller, 6–10 nm nanoparticles, higher number of atoms goes through the larger nanoparticles and induce the shape transformation while smaller nanoparticles retains spherical shape. Our study, however, shows that molten-ion-track-induced elongation can also be active process in the nuclear stopping regime. Further the dissolution of Au atoms into SiO_2_ matrix can take place through flow in the molten track and also from ballistic ejection due to nuclear energy loss from both the larger and smaller nanoparticles.

**Figure 5:**
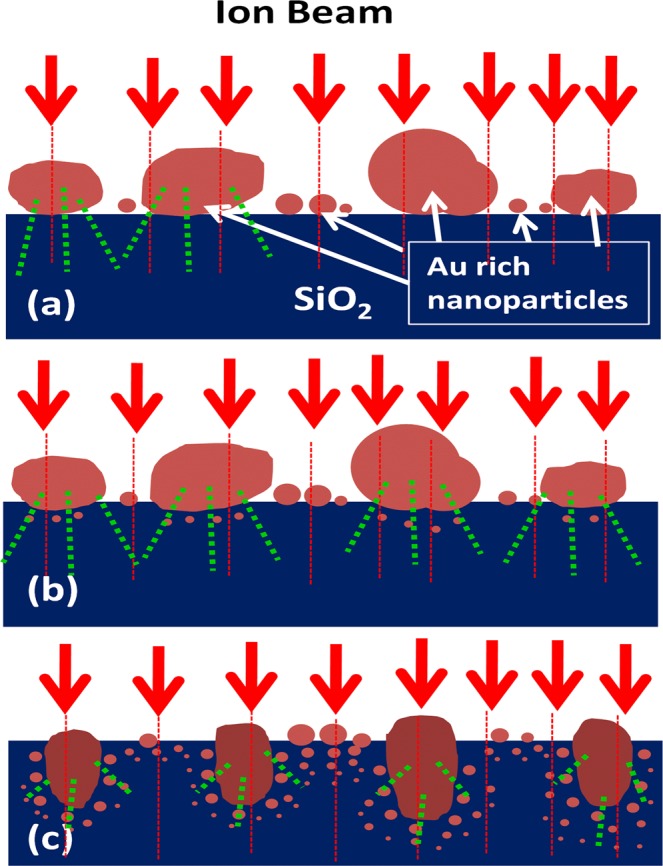
Schematic diagram of the nanoscale morphology evolution process (**a**) the Au-rich nanoparticles forms on SiO_2_ surface under irradiation. The red dashed lines through the nanoparticles signify the latent ion tracks formed in the nanoparticles and the substrate. The green dotted lines signify the ballistic ejection and dissolution of atoms from nanoparticles to the matrix due to nuclear collisions (**b**) nanoparticles get burrowed into the substrate to minimize surface free energy and new nanoparticles nucleate under the existing ones due to supersaturation of the matrix with dissolved Au toms (**c**) large nanoparticles gets elongated and satellite nanoparticles grow by material flow from supersaturated matrix to satellites.

Subsequent to burrowing, we observe nucleation and growth of small nanoparticles below the larger ones [Figs 3 and 4]and appearance of satellite nanoparticles around the elongated ones. Nucleation of metal nanoparticles in insulator matrix under ion irradiation was studies by several groups^42,47–51^. Nanoparticles nucleate in the matrix when the concentration of metal atoms (Au in present case)getting dissolved in the matrix from the primary nanoparticles by ballistic ejection or melting in ion track exceeds supersaturation limit. Under ion irradiation, the concentration of ejected atoms C^I^(R_0_) near the interface of a nanoparticle with the matrix of radius R_0_ is given by C^I^(R_0_) = C_∞_^I^ (1 + R_C_^I^/R_0_)^49^. Here C_∞_^I^ is the concentration of solutes near a planer interface, and R_C_^I^ is called capillarity length. The quantities depend upon distribution of ballistically ejected atoms due to nuclear collisions, ion flux and diffusion coefficient of the nanoparticle atoms in the matrix. Under ion irradiation, the capillarity length may become negative so that the concentration of atoms near bigger nanoparticles is higher and smaller nanoparticles grow at due to diffusional solute flow from bigger nanoparticles to smaller ones^42,47–50^. This mechanism of growth is known as inverse Owsald ripening. In our experiment, the smaller nanoparticles which nucleate below the bigger nanoparticles [Fig. 2(b)] clearly exhibits that Au concentration in SiO_2_ is over supersaturation limit due to dissolution by incident ions. The growth of nucleated nanoparticles with irradiation may proceed in different ways depending upon ion flux and irradiation temperature. A model by Heinig *et al*.^49^ predicted nanoparticle growth by inverse Oswald ripening at room temperature. Experimental studies by Rizza *et al*. also showed^42,47^ appearance of satellite nanoparticles around primary Au nanoparticles in SiO_2_ of diameter ~15 nm under 4 MeV Au ion irradiation. The diameters of these first generations of satellites increased with ion fluence while further generations of smaller satellites nucleated at increasing distance from the primary one. This was explained in terms of superaturation of SiO_2_ by ballistic ejection of atoms from primary nanoparticles where primary nanoparticle is a source of Au atoms where nucleation of further generation of satellites was related to partial dissolution of existing satellites under irradiation. Further, Ruffino *et al*. showed that Au nanoparticles can grow under irradiation of 200 keV Ar-ions ions at low flux of 1.4 μA^52^. In our experiment, the ion flux of 1.4 μA can be expected to lead to growth of nanoparticles. Recently Vu *et al*. has shown^53^ for 4 MeV Au-ion irradiation of 4 nm Au nanoparticles in SiO_2_ at 300 K that diffusion of solutes is larger than thermal diffusion and smaller nanoparticles may nucleate and grow under ion irradiation. In the present study, Fig. 3(b) which exhibits decreasing radii of the satellite nanoparticles from 2–3 nm close to the primary ones to <1 nm in the furthest region of ~15 nm from the elongated nanoparticles is quite consistent with the study of Rizza *et al*. and can be correlated to inverse Oswald ripening^47,48^. Observed evolution thus signify the fact that concentration of dissolved nanoparticles increase with fluence because Au atoms are knocked out from the larger nanoparticles and the satellite ones into the matrix. Further, growth of nanoparticles below the surface layer of 6–10 nm Au nanoparticles partially buried in SiO_2_ is also support the picture of continuous dissolution of Au atoms. Similar to satellite growth around elongated nanoparticles, diameters of nucleated nanoparticles close to the surface layer of existing nanoparticles are higher and decrease continuously towards bulk. It seems plausible that the nanoparticles nucleated at higher distances by partial dissolution of grown particles close to the surface layer. We summarise the structural evolution in the schematic diagram of Fig. 5(a–c). Au-rich nanoparticle array is formed on SiO_2_ by ion beam dewetting at the initial stage [Fig. 5(a)] where smaller nanoparticles are in between the larger nanoparticles. Under cumulative effect of ion irradiation material flow occurs within molten ion track going through the nanoparticles [shown as dashed red lines in Fig. 5(a)]. This causes two effects, namely burrowing of the nanoparticles into the matrix and elongation of large nanoparticles because they receive grater no of incident ions due to larger surface area [Fig. 5(b)]. At the same time, Au atoms are dissolved into SiO_2_ matrix by nuclear collision induced ballistic process [shown as dotted green lines in Fig. 5(a–c)] as well as flow in molten track. When the concentration of solute atoms goes over supersaturation, nanoparticles nucleate in SiO_2_ and grow by continuous supply of solutes under irradiation. Further generations of nanoparticles grow at larger distances from the primary ones due to partial dissolution of grown satellites [Fig. 5(b,c)].

**Figure 6:**
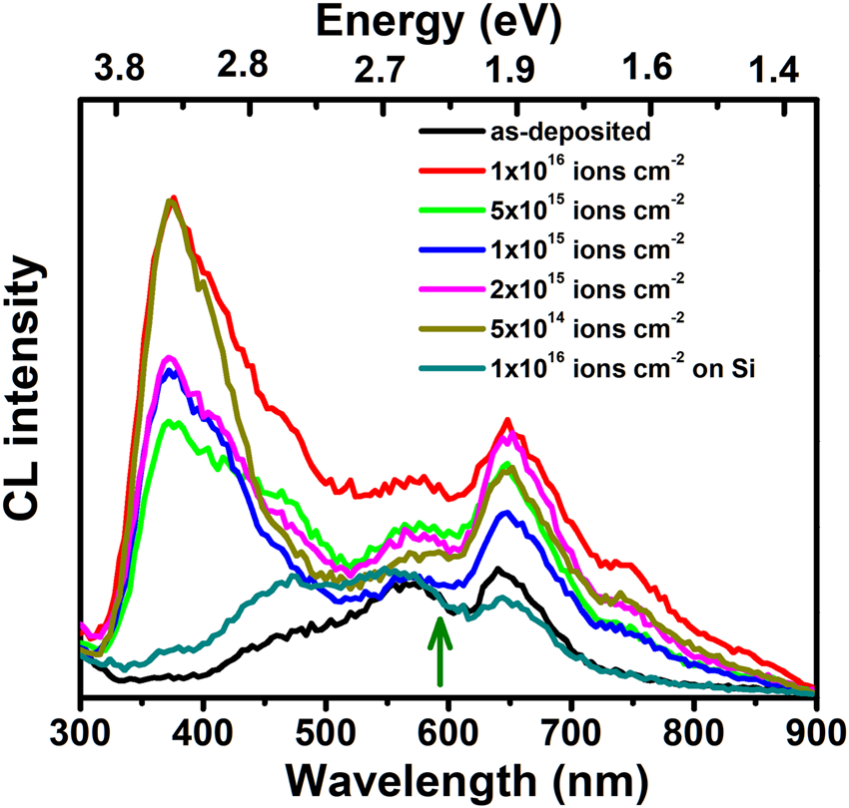
CL spectra from the irradiated samples comprising of nanoparticle array and an irradiated Si layer on SiO_2_. The fluences are indicated on the images. The green arrow shows SPR emission wavelength of Au nanoparticles.

**Figure 7:**
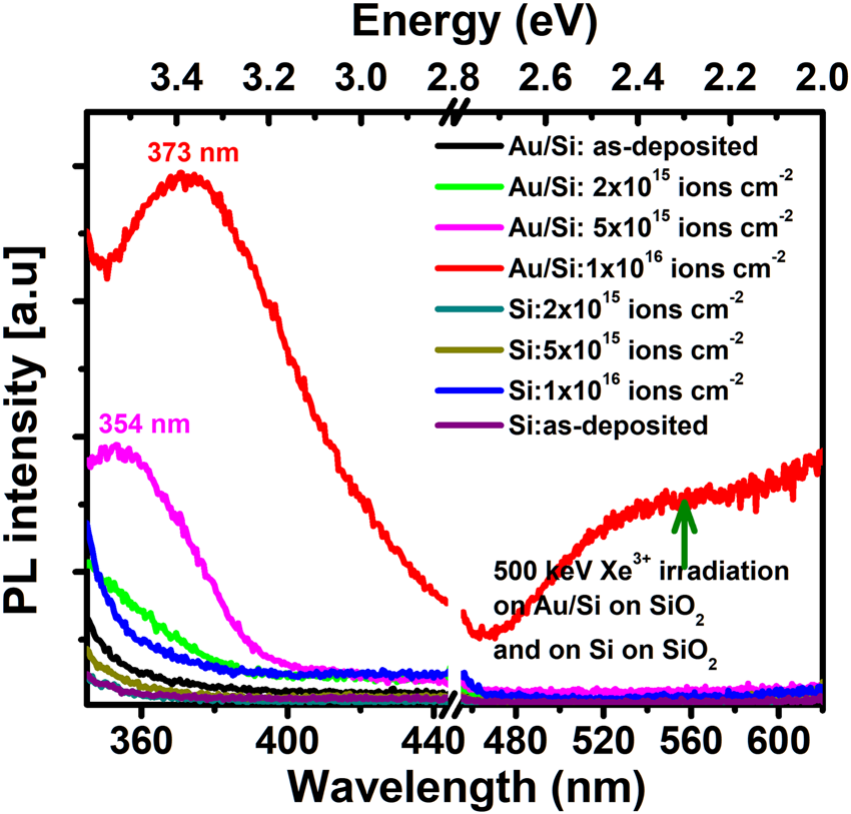
PL spectra from the irradiated samples comprising of nanoparticle array and an irradiated Si layer on SiO_2_. The fluences are indicated on the images. The green arrow shows SPR emission wavelength of Au nanoparticles.

Visible range luminescence of composite nano-dot array evolves with morphology as depicted in Fig. 6. It is to be noted here that intensity of CL emission cannot be correlated with any morphological or structural evolution since the setup had to be re-optimized for every sample. Two emission bands in the UV and visible region are prominent around 380 nm and 650 nm from the initial stage of nanostructural evolution, i.e., from fluence 5 × 10^14^ ions cm^−2^, where nanoscale islands are observed to grow on the surface [Fig. 1(a)]. In addition, existence of a peak around 475 nm can be traced in the spectra. A peak is present around 650 nm in the spectrum from the as-deposited sample although no emission band around 380 nm is observed. A small emission band is found around 575 nm in spectra from both the as-deposited and irradiated samples. Further, a band in the near infrared range with peak at around 750 nm is observed in the spectra of ion irradiated Au-Si bilayers. It should be noted here that no prominent emission at 380 nm is detected in the spectrum from the irradiated Si film on SiO_2_ although faint peaks are discernible at 475, 575 and 650 nm range. The photoluminescence spectra from the irradiated Au-Si samples are shown in Fig. 7. The PL spectra are consistent with CL, although detectable photoluminescence is obtained only for samples implanted to higher fluences, namely, 5 × 10^15^ and 1 × 10^16^ ions cm^−2^. The visible peaks are discernible at 354 and 373 nm respectively, which show a red shift with increasing fluence. It is also observed from Fig. 7 that the intensity of luminescence increases with fluence.

Luminescence of Au, Si, and SiO_2_ is correlated with the structural modification in the nanoscale and corresponding band structure^54,55^. We have seen that the nanostructures observed in the present experiment are dominatingly Au with AuSi phase present^27^. Thus the CL and PL from these composite nanostructures may originate from Au as well as from AuSi phase in the nanoscale. As for the UV band with peak around 380 nm, we rule out the CL/PL emission from SiO_2_ or ion irradiated Si/SiO_2_ since we do not detect any emission from such samples at this wavelength [Figs 6 and 7] and associate it with Au or AuSi composite nanostructure. The luminescence from Au nanoparticles is size dependent and may originate from different processes. In case of few atoms clusters, quantum confinement and modification of band structures of Au can give rise to luminescence in the UV to visible range, depending upon the cluster size^54,55^. On the other hand, in Au nanoparticles of dimensions varying from a few nm to tens of nanometers, PL is associated with radiative recombination of the electrons in the conduction band with holes in the *d*-band^55^. XTEM images of Figs 3(b) and 4(a–c) show a few small (<1 nm) satellite nanoparticles at the initial stage of evolution and growth of large number of satellite nanoparticles at the late stage, where the nanoparticle diameters decrease from 2–3 nm to sub-nm level as a function of distance from the core. Thus, the UV luminescence around 380 nm may originate from the Au clusters present in the SiO_2_ matrix^54,55^. In fact, PL emission at 3.22 eV was found to originate from Au_5_ clusters by Zheng *et al*.^55^ due to electronic transition in modified band structure of Au. In addition, a red-shift of PL peak with cluster size was also detected^55^. Thus the increased PL intensity and small red shift with fluence, as observed in Fig. 6, can be qualitatively understood in this framework as due to presence of few atom Au clusters in SiO_2_. It should be mentioned here that emission from clusters can red shift further in the wavelength range of 500–900 nm, although the such luminescence from clusters, in the 500–700 nm region, is difficult to distinguish from those of the defect centers (described below) for the present study. The peak around 750 nm in Fig. 6, discernible only in the spectra from irradiated Au-Si bilayers, i.e., where small Au clusters exists in SiO_2_, may originate from Au_23_ clusters^54^. Moreover, Liao *et al*. observed that for Au nanoparticles of diameter 15–200 nm embedded in SiO_2_ matrix, PL emission shows red shift from ~550 nm to 650 nm when the particle diameter varies from 36 nm −78 nm, whereas no further red shift was observed for larger particle diameters^56^. The emission process was attributed to recombination of *sp-*band electrons with *d*-band holes. Therefore, the emission peak at 750 nm does not seem to be from large Au nanoparticles observed in Figs 2(b) and 4(a–c), and further ascertains contribution of Au clusters in the luminescence observed in the present study. The PL in UV region, however, has also been observed in Au nanoparticles of higher dimension (>1–2 nm). Dhara *et al*. observed PL emission at 400 nm (3.1 eV) and 364 nm (3.4 eV) for Au nanoparticles in SiO_2_, of diameter varying from 4–9 nm with an average diameter 7.7 nm^57^. PL from the Au nanoparticles was explained by electronic transition from conduction band to *d*-band holes. We also observed Au satellite nanoparticles of diameter 2–3 nm in the regions close to the core ones. We therefore do not rule out contribution of bigger nanoparticles in the near UV luminescence in the present experiment. Regarding the luminescence in the visible range, a broad PL band in the range 500–620 nm was also observed by Sharma *et al*.^58^ for Au nanoparticle coated on SiO_2_. PL in 550 and 650 nm was also demonstrated for Au nanospheres and there dimmers by Huang *et al*.^59^ In both cases, PL was attributed to band transition in Au with consideration of the influence of plasmonic coupling due to Au SPR. In our study, however, we observe CL peak in the spectrum from the as deposited Au-Si bilayer on SiO_2_ and also for Si layer on SiO_2_ irradiated to the highest fluence. Therefore, the bands observed at in 475, 575, and 650 nm seem to be mainly related with defects in SiO_2_, although contribution from larger Au nanoparticles may be present. In fact, PL peak observed at 460 nm (2.7 eV) was attributed to E´ centers in pristine and irradiated SiO_2_ by Warang *et al*.^60^. A band around 1.9 eV (650 nm) was ascribed to non bridging oxygen hole centers (≡Si-O) in SiO_2_ by Hu *et al*.^61^ and a peak observed at 580 nm was correlated with defects in SiO_2_ by Huang *et al*.^62^. Following them, we attribute the bands in 475–650 nm mainly to luminescent defect centers in SiO_2_ with possible contribution from Au nanoparticles. The peak at 475 nm, in particular, becomes sharper in case of irradiated Au-Si and Si films on SiO_2_, indicating increase in defects like E´ centers which was previously observed by Warang *et al*.^60^. On the other hand, a peak 628 nm and 683 nm was attributed to AuSi_x_ nanostructure by Huang *et al*.^62^ and Wu *et al*.^63^ respectively, which can also be present in our case because XPS observation revealed existence of Au-silicides in the nanostructures.

## Summary

In summary, we have investigated the development of Au rich nanoparticle morphology in SiO_2_ due to ion irradiation on a bilayer of Au-Si from the initial low fluence to a final high fluence regime. The SEM and XTEM results demonstrate successive stages of nanoscale morphological evolution starting from formation of a nano-array on SiO_2_ surface where the main driving force is ion-beam-dewetting. Through numerical simulation based upon a thermal spike model, we have demonstrated formation of molten ion-tracks in Au and SiO_2_ which results in dewetting. The simulation result, combined with XTEM results indicates that dewetting is initiated by formation of dry patches. We have shown gradual burrowing of the nanoparticles into SiO_2_ subsequent to formation of nanoparticle array on the surface and simultaneous elongation of large nanoparticles in the direction of incident ions, where the required atomic mobility is provided by ion-induced latent ion track formation. Our experimental findings also confirm that the burrowing is caused by tendency of the system towards minimization of surface energy. Growth of satellite nanoparticles of varying diameter around the embedded larger particles by inverse Oswald ripening is shown to accompany the burrowing effect. We have also shown UV and Visible range luminescence in the ion-beam-synthesized nanostructured materials. The observed luminescence is understood to originate from nanoscale Au particles in SiO_2_ at different stages of ion irradiation, which shows the potential of ion beam processing in synthesis of UV-infrared luminescent embedded nanostructures. Since protrusion of developed nanostructures over the matrix has been shown, our results are further promising for technological application such as surface enhanced Raman scattering.
